# Non-Destructive Assessment of Masonry Pillars using Ultrasonic Tomography

**DOI:** 10.3390/ma11122543

**Published:** 2018-12-13

**Authors:** Monika Zielińska, Magdalena Rucka

**Affiliations:** 1 Department of Technical Fundamentals of Architectural Design, Faculty of Architecture, Gdansk University of Technology, Narutowicza 11/12, 80-233 Gdansk, Poland; monika.zielinska@pg.edu.pl; 2 Department of Mechanics of Materials and Structures, Faculty of Civil and Environmental Engineering, Gdansk University of Technology, Narutowicza 11/12, 80-233 Gdansk, Poland

**Keywords:** non-destructive testing, masonry structures, strengthening, ultrasonic tomography, adhesion assessment

## Abstract

In this paper, a condition assessment of masonry pillars is presented. Non-destructive tests were performed on an intact pillar as well as three pillars with internal inclusions in the form of a hole, a steel bar grouted by gypsum mortar, and a steel bar grouted by cement mortar. The inspection utilized ultrasonic stress waves and the reconstruction of the velocity distribution was performed by means of computed tomography. The results showed the possibilities of tomographic imaging in characterizing the internal structure of pillars. Particular attention was paid to the assessment of the adhesive connection between a steel reinforcing bar, embedded inside pillars, and the surrounding pillar body.

## 1. Introduction

A significant part of engineering structures consists of masonry objects. Historic buildings are usually made of bricks or stones [1,2] while to make contemporary objects, both ceramic elements and blocks of autoclaved aerated concrete are used [3]. The technical condition of masonry structures requires a careful quality assessment and often intervention, enabling their further proper functioning, due to the influence of atmospheric conditions, excessive loads and processes of the natural ageing. Properly carried out works, aimed at strengthening, repairing or maintaining masonry, should be preceded by a precise diagnosis process. In general, two main diagnostic approaches are possible: invasive and non-invasive [4]. In destructive testing (DT), material samples acquired from an object are destroyed to evaluate their mechanical properties. Particularly important in the diagnostics of engineering structures are non-destructive testing (NDT) methods, because they do not violate the integrity of tested objects. In recent years, various non-destructive techniques dedicated to masonry structures have been developed, including ultrasonic methods, ground penetrating radar, thermography or acoustic emissions (e.g., [1,5,6,7,8,9,10]).

The basic methods for strengthening masonry structures are total or partial brick replacement and the introduction of new reinforcing elements. The brick replacing technique relies on substituting old, degraded material with new material. This method significantly affects the structure of the object, and it is not recommended for works carried out on objects of cultural heritage, where any interference with the historic substance should be avoided. The structural efficiency of masonry can be also increased by introducing new strengthening elements (e.g., [11,12]). Reinforced concrete elements, introduced in the form of columns and beams [13] or jackets [14] constitute a substitute supporting structure. Additional steel (or composite) elements are usually used as grouted anchors or tie-rods (e.g., [15,16,17,18]) as well as composite jackets made of fabric-reinforced cementitious matrix [19] or fiber-reinforced polymers [20,21]. If a replacement technique is used, the adhesion of an old wall to a new part is not as important, because usually the new wall has only the role of filling, and in this case, it is completely lightened. However, the adhesive connection is very important when steel elements in the form of bars are used, because their main purpose is to transfer stresses to the masonry. Incorrect anchoring of the steel elements, or damage developing at the interface between two materials, i.e., steel and mortar, may be the reason why the strengthening method does not fulfil its assumed function. Therefore, the non-destructive evaluation of the adhesive connection is crucial for properly conducting the repairing process.

In this work, an evaluation of masonry pillars is presented. Particular attention was paid to the condition assessment of the adhesive connection between a steel reinforcing bar embedded inside pillars and the surrounding pillar body. The research was carried out using ultrasound waves and tomography imaging. Ultrasound tomography is one of the more developed imaging methods, utilizing the properties of elastic waves. Many previous works concern Lamb-wave tomography for damage detection in metal or composite thin plates. Rao et al. [22] performed a study on the online corrosion monitoring of a steel plate and thickness reconstruction of the corrosion damage. Zhao et al. [23] investigated damage imaging in aluminum plates with an artificial thinning area. Leonard et al. [24] studied Lamb-wave tomography in both aluminum and composite plates, with defects of various sizes and thicknesses. A structural health monitoring system of composite plates with through-thickness holes was presented by Prasad et al. [25]. Ultrasound tomography was also widely used for defect imaging in concrete structures (e.g., [26,27]). Martin et al. [26] examined post-tensioned concrete beams. They identified both the location of ducts and the voiding in ducts. Chai et al. [28] developed attenuation tomography for visualizing defects in a concrete slab. Aggelis et al. [29] applied numerical simulations of wave propagation to investigate the possibility of detecting different types of inhomogeneities in concrete. Schabowicz and Suvorov [30] described an ultrasonic tomogram, equipped with a multi-element antenna array, and its application for the estimation of the thickness of concrete and the localization of flawed zones. Schabowicz [31] presented the one-sided non-destructive testing of concrete cubic specimens and a foundation concrete slab using ultrasonic tomography. Haach and Ramirez [32] analyzed different arrangements of transducers in a study on the detection of cylindrical polystyrene blocks in concrete prismatic specimens. Choi and Popovics [33] compared one-sided imaging with through-thickness tomography using the example of highly reinforced concrete elements with internal defects. Integrated ultrasonic tomography and a 3D computer vision technique was developed by Choi et al. [34]. They obtained volumetric internal images to detect defects within concrete. Chai et al. [27] identified the tendon duct filling, as well as honeycomb defects, in concrete specimens. In the case of masonry structures, ultrasonic tomography was used to assess the general condition or to evaluate the effectiveness of the conducted repair. Schuller et al. [35] performed a velocity reconstruction in a wall specimen made of bricks. Binda, Saisi, and Zanzi [36] examined the stone pillars of the temple of S. Nicolò l’Arena using sonic tests. They obtained maps of the velocity distribution that enabled the presence of the different building techniques applied during the erection of the pillars to be recognized. The detection of voids in laboratory masonry specimens was studied by Paasche et al. [7]. They applied ultrasonic tomography and compared the results with those of ground penetrating radar tomography. Pérez-Gracia et al. [37] determined the velocity distribution in columns of the Mallorca Cathedral. Santos-Assunçao et al. [9] evaluated laboratory models of masonry columns. Changes in the wave velocity in tomography maps were identified as damages or changes in the bricks and mortar. The literature on the assessment of reinforced masonry structures is rather limited.

This study presents a comprehensive condition assessment of masonry pillars. Experimental and numerical investigations were performed on an intact pillar as well as three pillars with internal inclusions in the form of a hole, a steel bar grouted by gypsum mortar, and a steel bar grouted by cement mortar. The inspection was conducted using ultrasonic waves and computed tomography. The investigations focused on the characterization of the internal structure of the pillars by velocity reconstruction in the examined cross-section. The influence of different numbers of pixels and different configurations of paths on tomographic velocity reconstruction was studied.

## 2. Theoretical Background of Ultrasonic Tomography

**Figure 1:**
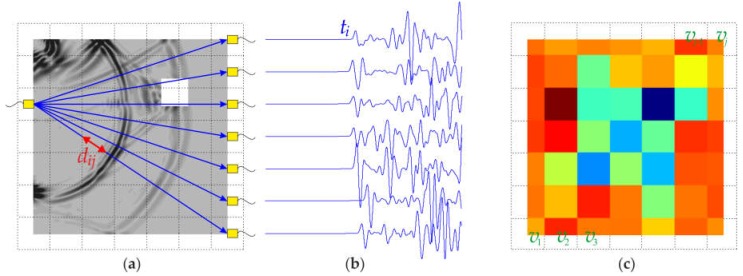
Schematic diagram of ultrasound tomography: (**a**) cross-section divided into pixels, with an indicated transmission point, receivers and a simulated wave field; (**b**) wave propagation signals; (**c**) tomographic image.

Ultrasonic tomography imaging allows the internal structure of an investigated object to be reconstructed by a set of projections through the sample in many different directions. A schematic diagram of ultrasound tomography is shown in Figure 1. At first, the tested specimen must be divided into small, geometrical cells, called pixels (marked with dotted lines in Figure 1a). Each pixel in the image represents a discrete area in the sample, and it is associated with an intensity value. The conducted calculations enable a quantitative description of each pixel, based on its physical characteristics, such as the wave propagation velocity. The number of pixels can be changed, enabling the study of the effect of spatial resolution on the quality of the obtained image.

In this study, through-transmission tomography was applied (cf. [29,32,33,34,27]). The tomographic procedure is based on elastic waves passing through the tested specimen, from the transmitter (T) to receivers (R) (Figure 1b). The image reconstruction is performed based on the information obtained from the received pulses. Typically, this information is the time-of-flight (TOF), measured along many ray paths. Based on the known geometry of the specimen, the average velocity of wave propagation can be determined. This velocity depends on the mass density, modulus of elasticity and Poisson’s ratio, characterizing the material inside the tested specimen. Any obstacle present in the path of the travelling wave results in a change of the propagation time. Defects, in the form of air voids or cracks on the wave path, cause a delay in the ray reaching the receiver. On the other hand, inclusions made of materials characterized by higher propagation velocities than the neighboring medium lead to an increase of the total velocity along all the rays passing through this inclusion.

The TOF between the transmitter and receiver can be represented by a line integral of the transition time distribution along the propagation way *w*:

(1) $$t=\int_{T}^{R}dt=\int_{T}^{R}sdw=\int_{T}^{R}\frac{1}{v}dw,$$

where *v* denotes the average velocity, and *s* denotes the slowness, which is the inverse of the velocity. Therefore, by measuring the direct wave passing time, it is possible to determine the distribution of local velocities in the tested cross-section. The reconstitution of the velocity profile $v_{j}$ for each cell in the plane of the transition of wave rays from the transmitter to the receiver can be performed based on the following formula:

(2) $$t_{i}=\sum_{j=1}^{n}w_{ij}s_{j},\;i=1,2,3,\ldots ,m,\;j=1,2,3,\ldots ,n,$$

where *m* is the number of measurement paths (rays), *n* is the number of cells (pixels), $w_{ij}$ denotes the transition way of the *i*-ray through the *j*-pixel, $t_{i}$ denotes the transition time of the P-wave, between the transmitter and receiver along the *i*-ray, and $s_{j}$ is the slowness at pixel *j*, whose inverse is the velocity $v_{j}$, i.e., $v_{j}=1/s_{j}$. It is assumed that the value of the velocity $v_{j}$ in individual cells is constant.

Equation (2) can be expressed in matrix form, with known matrices *t* and *w* (representing the passage path through a given cell and the transition time along a given path, respectively) as well as the unknown matrix *s* (slowness):

(3) $$\left[\begin{array}{c} t_{1}\\ t_{2}\\ t_{3}\\ \vdots \\ t_{i} \end{array}\right]=\left[\begin{array}{ccccc} w_{11} & w_{12} & w_{13} & \cdots & w_{1j}\\ w_{21} & w_{22} & w_{23} & \cdots & w_{2j}\\ w_{31} & w_{32} & w_{33} & \cdots & w_{3j}\\ \vdots & \vdots & \vdots & \ddots & \vdots \\ w_{i1} & w_{i2} & w_{i3} & \cdots & w_{ij} \end{array}\right]\left[\begin{array}{c} s_{1}\\ s_{2}\\ s_{3}\\ \vdots \\ s_{j} \end{array}\right].$$

In computed tomography, the above system of equations is usually overdetermined or underdetermined, so the problem, described by Equation (3), is ill posed. It can be efficiently solved using the algebraic reconstruction technique (ART) [38]. In the first step of the calculations, each cell is assigned the same slowness (inverse of the velocity value, equal to the average velocity of the wave propagation in the examined material). In this way, an initial image is created. Next, the iteration process is started, and corrections are calculated according to the relation [39]:

(4) $$s_{j}^{\left(k\right)}=s_{j}^{\left(k-1\right)}+\frac{w_{ij}\Delta t_{i}}{\sum_{j=1}^{n}w_{ij}^{2}},$$

where $\Delta t_{i}$ is the difference between the time of the original projection and the rebuilding time. The system of equations is iteratively solved until the reconstructed travel time reaches the measured travel time, with an established error.

## 3. Materials and Methods

### 3.1. Description of Specimens

**Figure 2:**
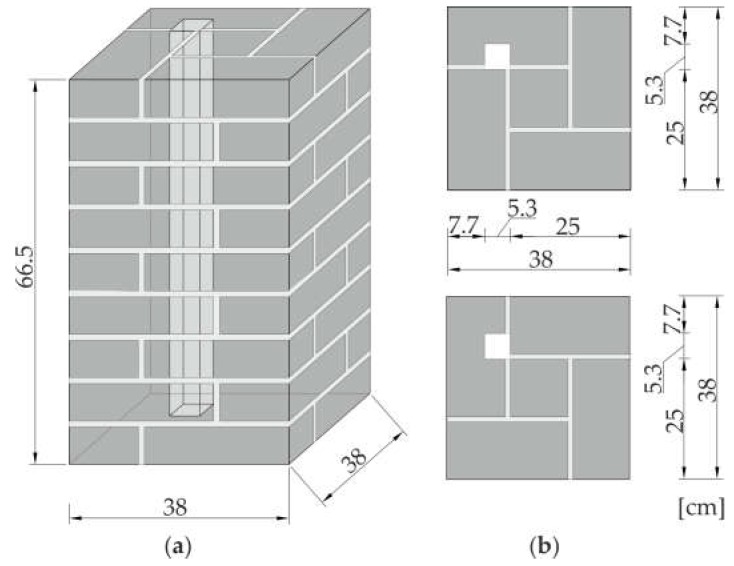
Geometry of the tested pillars: (**a**) 3D view; (**b**) plane view of even and odd layers.

A masonry pillar was used as a testing object. The pillar was manufactured using solid bricks, with dimensions of 25 cm × 12 cm × 6.5 cm. Joints between bricks were 1 cm thick and were filled with gauged (cement-lime) mortar. Nine brick layers were used to build the specimen. The model had a length of 66.5 cm and cross-section dimensions of 38 cm × 38 cm. The geometry of the pillar and a view of the even and odd layers are shown in Figure 2.

**Figure 3:**
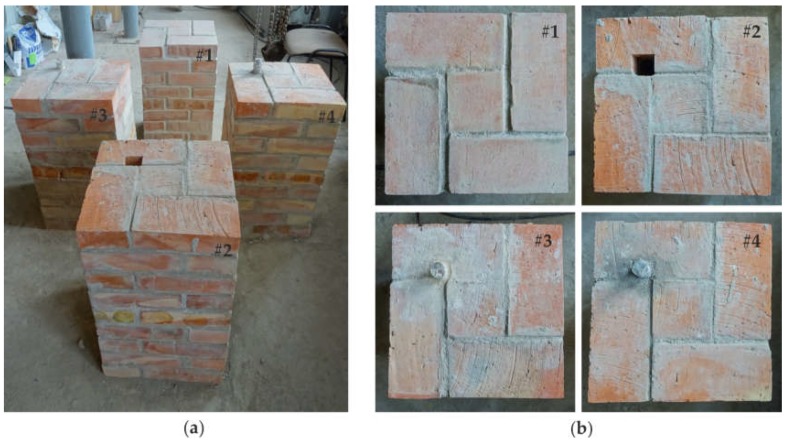
Photographs of laboratory models of the brick pillars: (**a**) overall view; (**b**) plane views of specimens #1 to #4.

The test specimens included one pristine pillar and three pillars with inclusions (Figure 3). The first pillar (#1) was prepared as a reference model. Pillars #2, #3 and #4 had a square hole through the entire height of the specimen. The hole, with dimensions of 5.3 cm × 5.3 cm, was situated at a distance of 7.7 cm from the pillar edges (Figure 2b). The hole in pillar #2 remained empty, while in the case of pillars #3 and #4, an anchor was mounted in it. The threaded steel bar had a diameter of 32 cm, and was grouted using two types of mortar. In pillar #3, the anchor was mounted using gypsum mortar to represent a weak bond, while in the case of pillar #4, cement mortar was used to represent a strong bond.

### 3.2. Identification of the Material Parameters

The tests were carried out on a single brick as well as on cubes made of cement mortar, gypsum mortar and gauged mortar, with dimensions of 10 × 10 × 10 cm^3^. Each sample was measured and weighted, and then the mass density *ρ* was calculated. The Young’s modulus *E* of brick and mortar was determined by a non-destructive approach using the ultrasonic pulse velocity (UPV) method ([40,41,42]). This approach was chosen because in this study a dynamic analysis was carried out in the ultrasonic frequency, which requires the identification of the dynamic modulus. In each sample, the transmission time of the P-wave was measured, and the P-wave velocity $c_{p}$ was calculated. The Poisson’s ratio ν was assumed, following Alberto et al. [43], as 0.15 for mortar and 0.2 for brick. Finally, the Young’s modulus was calculated according to the following equation:

(5) $$c_{p}=\sqrt{\frac{E(1-\nu )}{\rho (1+\nu )(1-2\nu )}}.$$

The results of the identified parameters are given in Table 1.

### 3.3. Experimental Setup

**Figure 4:**
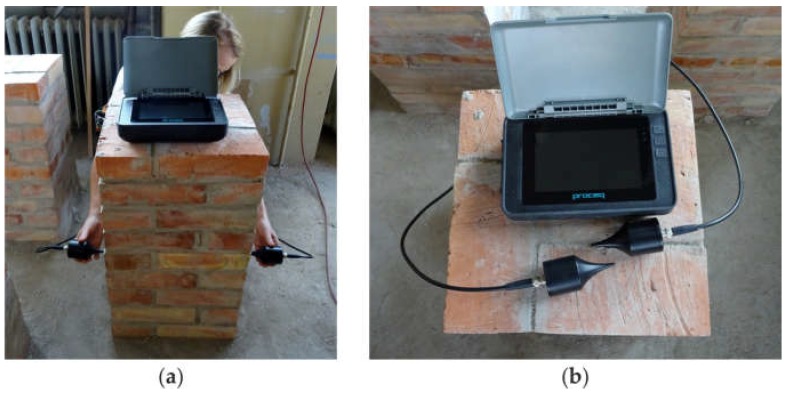
Experimental setup: (**a**) testing in through transmission mode; (**b**) registration unit and exponential transducers.

After 28 days, the masonry pillars were subjected to ultrasonic tests. The velocity of the stress waves was measured using the ultrasonic pulse velocity method. The PUNDIT PL-200 instrument (Proceq SA, Schwerzenbach, Switzerland) was utilized for measurements of the ultrasonic pulse velocity. Two exponential transducers of 54 kHz were used in the through transmission mode, and dry coupling was applied. The experimental setup is shown in Figure 4.

**Figure 5:**
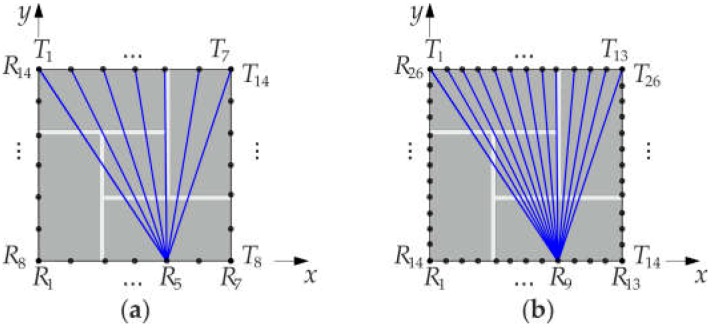
Configuration of the measurement points: (**a**) configuration #A (transmission of waves at points *T*_1_-*T*_14_ and sensing of waves at points *R*_1_-*R*_14_), (**b**) configuration #B (transmission of waves at points *T*_1_-*T*_26_ and sensing of waves at points *R*_1_-*R*_26_).

Measurements were carried out on the middle (i.e., 5th) layer of bricks. Two experiments were conducted. In the first test (configuration #A), 7 measurement points were distributed at each pillar edge, while in the second test (configuration #B), measurements were taken at 13 points. The configurations of the measurement points are given in Figure 5. Transmitting points are denoted by T, and receiving points by R. The receiving transducer was set at a given point, and then the transmitting transducer was moved from the first to the last point on the opposite wall. There were 14 transmitting/receiving points for configuration #A and 23 transmitting/receiving points for configuration #B.

### 3.4. FEM Modeling

Numerical analyses of the stress wave propagation were carried out using the finite element method (FEM) in the Abaqus/Explicit. A two-dimensional model of the fifth layer of the pillar was developed using four-node plane strain elements, with reduced integration (CPE4R). The size of all elements in the model was 1 mm × 1 mm. The material of the pillar was assumed to have linear elastic behavior. The material properties of the bricks and mortar were determined experimentally (Table 1), and for the steel, the following parameters were adopted: *E* = 200 GPa, *ρ* = 7850 kg/m^3^, *v* = 0.3. The connections between the bricks and mortar as well as between the mortar and steel were modelled as a tie constraint. The boundary conditions were assumed to be free on all edges. The excitation signal was a one-cycle sine wave of 54 kHz frequency, modulated by the Hann window. The size of the integration step was 10^−7^ s. The output acceleration signals were recorded at the same points as in the experimental investigations (Figure 5).

### 3.5. Configuration of Pixels and Paths

**Figure 6:**
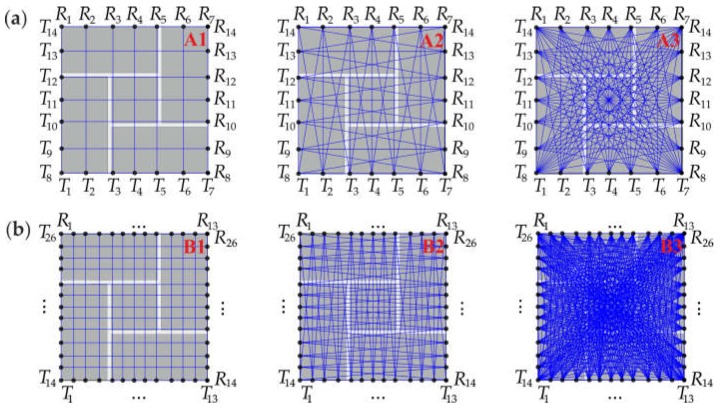
Configuration of paths: **(a**) configuration #A (#A1, #A2 and #A3); (**b**) configuration #B (#B1, #B2 and #B3).

The acquired experimental and numerical signals were processed to obtain tomography images. At first, the cross-section of the pillar was divided into 49 pixels, in the case of configuration #A, and 169 pixels, in the case of configuration #B. Data analysis was conducted in three stages, with different arrangements of pats (Figure 6). In the first stage, paths were assumed only between opposite transmitting/receiving points. There were 14 paths in configuration #A.1 and 26 paths in configuration #B.1. In the second stage, from each transmitting point, two or three paths were considered, giving 38 paths in configuration #A.2 and 74 paths in configuration #B.2. The third stage took into account seven paths from each transmitting point. As a result, 98 paths were traced in configuration #A.3 and 338 paths in configuration #B.3.

## 4. Results and Discussion

**Figure 7:**
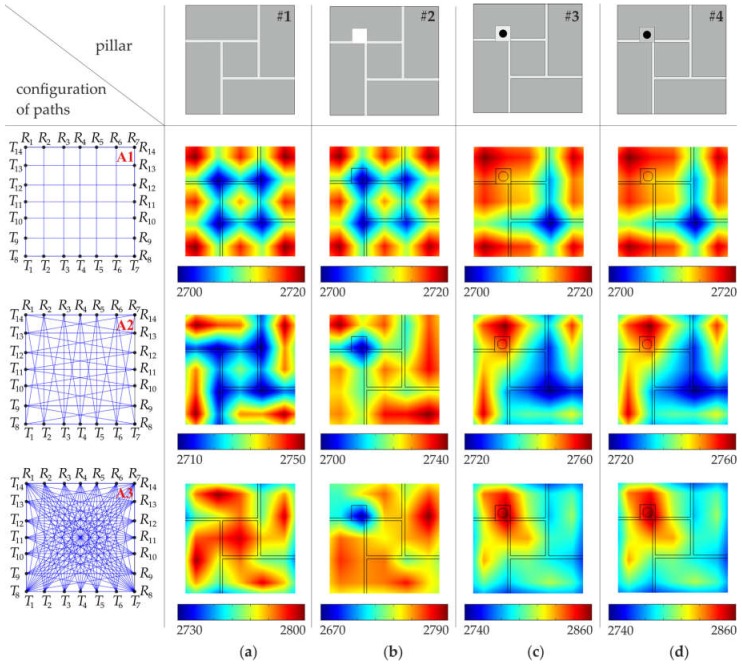
Tomographic velocity images (values in (m/s)), with a division into 49 pixels (configuration #A), obtained from FEM signals: (**a**) intact pillar; (**b**) pillar with the square hole; (**c**) pillar with the steel bar grouted by means of gypsum mortar; (**d**) pillar with the steel bar grouted by means of cement mortar.

**Figure 8:**
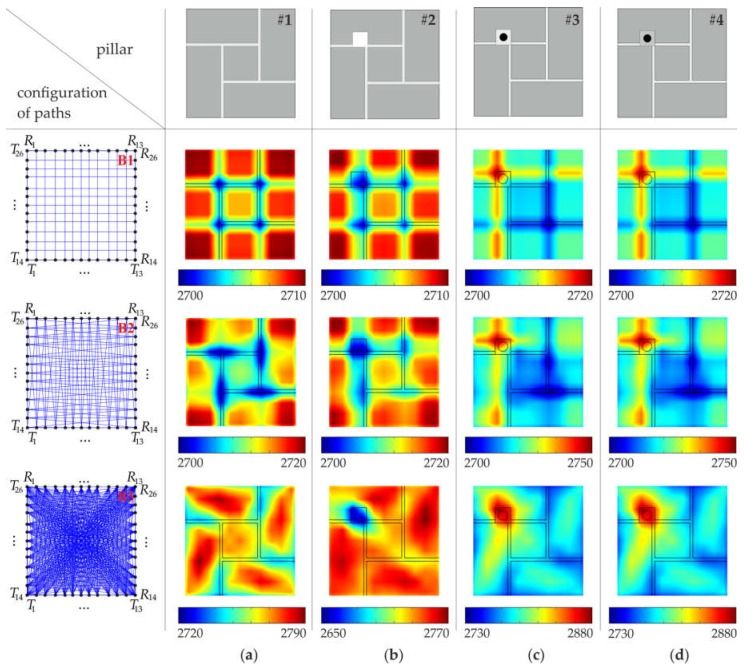
Tomographic velocity images (values in (m/s)), with a division into 169 pixels (configuration #B), obtained from FEM signals: (**a**) intact pillar; (**b**) pillar with the square hole; (**c**) pillar with the steel bar grouted by means of gypsum mortar; (**d**) pillar with the steel bar grouted by means of cement mortar.

Ultrasound tomography images, determined based on numerical FEM signals, are shown in Figure 7 and Figure 8, with a division into 49 and 169 pixels, respectively (see Section 3.4). In general, the increase in the number of transmitters, as well as the increase in the number of paths, improved the resolution of the velocity maps and provided a more precise image of the internal structure of the pillars.

Tomograms for the intact pillar (#1) enabled the identification of the arrangement of joints (Figure 7a and Figure 8a). Joints made of cement-lime mortar were characterized by a lower wave propagation velocity than the brick itself. The impact of the existence of joints on the tomographic images was visible, regardless of the number of transmitters used and the number of measurement paths. However, maps created based on the simplest configurations of paths (#A.1, #B.1) did not give satisfactory results regarding precise joint localization. The use of vertical and horizontal wave paths enabled only the identification of the directions of the mortar joints. More accurate tomographic images were obtained when a larger amount of measurement data was collected. Increasing the number of paths (configurations #A.2–A.3 and #B.2–B.3) allowed the position of joints in the considered cross-section and the exact brick arrangement pattern to be determined.

Tests conducted for the pillar with the hole (pillar #2) allowed its position to be estimated, but only when a sufficiently large number of ray paths was used (Figure 7b and Figure 8b). In the case of configurations of vertical and horizontal paths (configurations #A.1 and #B.1), the obtained tomographic images were very similar to those obtained for the intact pillar. To obtain the precise localization of the hole, the configuration, including all paths between the transmitting point and receiving points, were required. For such configurations (#A.3 and #B.3), a clear area, with a lower value of the velocity, can be observed. It was also possible to identify the arrangement of mortar joints, especially in the case of the image divided into 169 pixels.

The results for the pillars with the steel bar grouted by means of gypsum cement mortar are shown in Figure 7c,d (49 pixels) and Figure 8c,d (169 pixels). Tomographic velocity images of the simplest paths differed considerably between configuration #A.1 and #B.1. In the case of configuration #A.1, no path crossed the steel bar, therefore, it was not possible to localize the bar. However, since two paths passed directly along the joints, two straight lines with lower velocities appeared in the map, indicating the direction of the mortar joints. When 26 instead of 14 horizontal and vertical paths were used, two perpendicular paths crossed the steel bar. Therefore, for configuration #B.1, two longitudinal areas with a higher velocity appeared on the maps. These lines were the effect of blurring, which occurs in linear tomography. They indicated the presence of the bar but did not allow for its exact localization. The precise localization was achieved after considering more paths crossing the bar (configurations #A.3 and B.3). As a result, a distinct area with higher velocity values can be observed around the bar position. It should also be noted that the maps for pillars #3 and #4 are almost the same. This means that the main influence on the velocity distribution was the presence of the bar, and therefore, it was not possible to determine the type of mortar (gypsum or cement) used.

The quantitative analysis of the wave propagation values through pillars along many different directions, is given in Table 2 and Table 3. For each configuration of paths (#A.1–A.3 and #B.1–B.3), the velocity values were calculated along all rays, and then the minimum and maximum values as well as the mean value of the wave velocity were listed. Moreover, measures of variability, i.e., the standard deviation (SD) and coefficient of variation (CV), were calculated. The average values of the velocity of the particular arrangements were close to each other and ranged from 2719.18 m/s to 2745.48 m/s. The standard deviation ranged from 17.2 m/s to 29 m/s, and the coefficient of variation ranged from 0.63% to 1.07%. This indicates a small variation in the speed of wave propagation along the considered paths. Due to the idealized connections between mortar and bricks, implemented in FEM models, the velocity variations were caused only by internal inclusions.

The conducted research also allowed the impact of the number of joints along the wave path on the velocity of propagating ultrasonic waves to be determined. This effect was investigated for each of the four pillars (#1–#4), taking into account the results obtained for arrangement #B. While checking the influence of the number of joints on the wave velocities, paths crossing the hole or the bar as well as the paths along the pillar edges were omitted. The velocity of the wave passing through one joint was calculated as the mean obtained between points *T*_10_-*R*_10_, *T*_11_-*R*_11_, *T*_12_-*R*_12_, *T*_15_-*R*_15_, *T*_16_-*R*_16_, *T*_17_-*R*_17_. The velocity of the wave passing through two joints was calculated as the mean obtained between points *T*_6_-*R*_6_, *T*_7_-*R*_7_, *T*_8_-*R*_8_, *T*_19_-*R*_19_, *T*_20_-*R*_20_, *T*_21_-*R*_21_, and finally, for calculations concerning three joints, the data obtained between points *T*_1_-*R*_11_ and *T*_3_-*R*_13_ were used. The results of the obtained average propagation velocities are summarized in Table 4. The velocities obtained for each pillar are almost the same, because no considered path crossed internal inclusions. However, the difference between the velocity of the waves passing through one, two, and three joints was observed. The velocity value decreased with the increase of the number of joints on the wave path.

**Figure 9:**
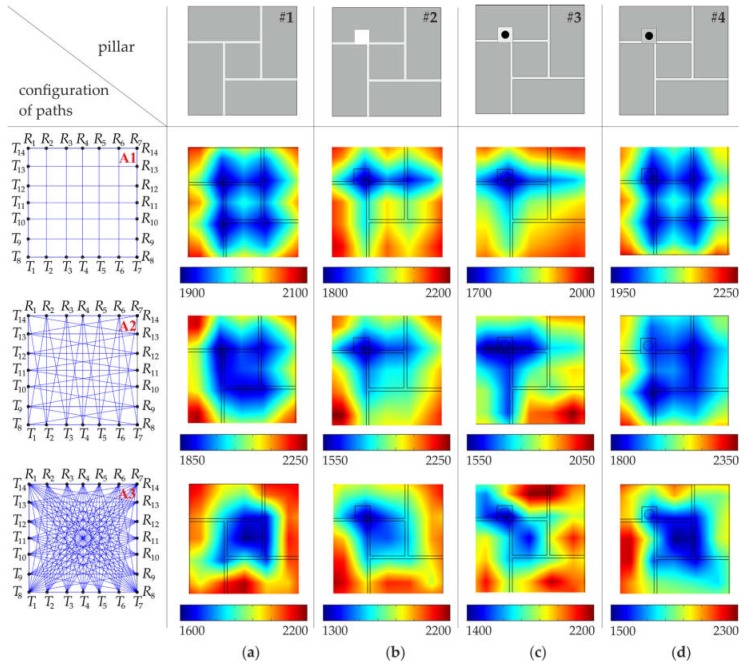
Tomographic velocity images (values in (m/s)), with a division into 49 pixels (configuration #A), obtained from experimental signals: (**a**) intact pillar; (**b**) pillar with the square hole; (**c**) pillar with the steel bar grouted by means of gypsum mortar; (**d**) pillar with the steel bar grouted by means of cement mortar.

**Figure 10:**
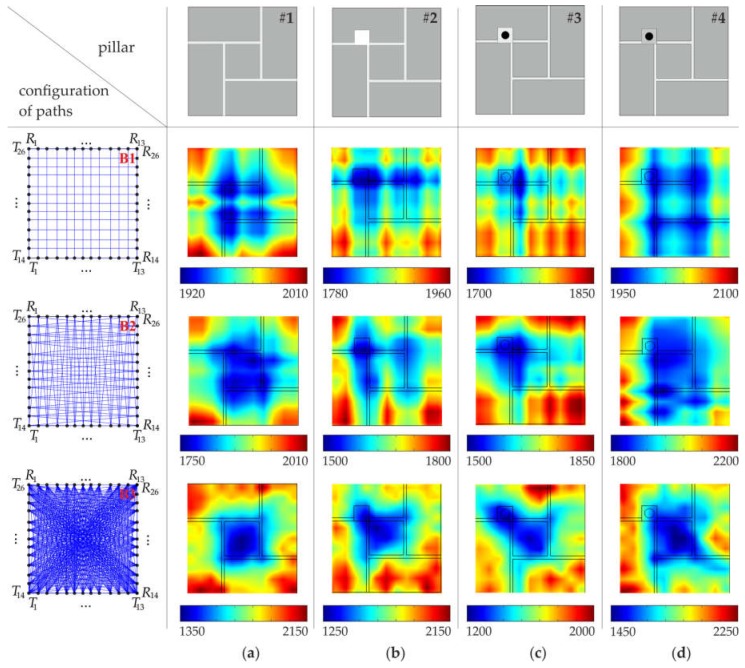
Tomographic velocity images (values in (m/s)), with a division into 169 pixels (configuration #B), obtained from experimental signals: (**a**) intact pillar; (**b**) pillar with the square hole; (**c**) pillar with the steel bar grouted by means of gypsum mortar; (**d**) pillar with the steel bar grouted by means of cement mortar.

The experimental tomographic images are shown in Figure 9 and Figure 10, with a division into 49 and 169 pixels, respectively (see Section 3.4). Contrary to numerical maps, the quality of experimental images did not increase with the increase of the number of pixels or paths used. In real masonry elements, connections between mortar and bricks are not ideal. Air voids, pores and a lack of adhesion between a brick and mortar may occur. The heterogeneous nature of the tested specimens as well as the imperfections of the connections between bricks and mortar caused a strong dissipation of the energy of propagating waves and consequently affected the quality of the obtained tomography images.

Measurements made for the intact pillar (#1) using only vertical and horizontal paths revealed lower propagation velocities at joint intersections (Figure 9a and Figure 10a). With the increase of the number of paths, for configurations #A.3 and #B.3 a zone with lower velocity values appeared in the central part of the pillar due to the accumulation of joints and paths crossing them.

Maps of the pillar with the hole are shown in Figure 9b and Figure 10b. The hole unambiguously disturbed the wave transition. The location of the hole can be indicated as the area with lower velocity values. The identification of the position of the hole was clearest in the case of vertical and horizontal rays (#A.1 and #B.1), however, it was affected by the blurring effect. As in the case of the intact pillar, the accumulation of lower velocities can be seen for configurations #A.2, #A.3 and #B.2, #B.3.

The maps obtained for the pillar with the bar grouted by gypsum mortar (#3) revealed similar patterns as those for the pillar with the hole (Figure 9c and Figure 10c). This may indicate poor adhesion between the bar and the gypsum mortar. On the other hand, the tomographic velocity images for the pillar with the bar grouted by cement mortar (#4) were very similar to those obtained for the intact pillar (Figure 9c and Figure 10c). This means that the connection between the bar and gypsum mortar was of a good quality, and strong adhesion occurred.

The minimum, maximum and average velocities of ultrasonic waves, based on experimental tests for each configuration of paths (#A.1–A.3 and #B.1–B.3), are shown in Table 5 and Table 6. The average values of the velocity for the particular pillars varied from 1635.34 m/s to 2163.11 m/s. The velocities for pillars #1 and #4 were higher than those for pillars #2 and #3, which proved weak adhesion in pillar #3 and strong adhesion in pillar #4. The standard deviation varied from 254.4 m/s to 465.7 m/s, and the coefficient of variation varied from 13.42% to 22.4%. These values were approximately 10–20 times larger than those obtained from the FEM simulations. At the same time, the experimental velocities appeared to be smaller than the numerical ones. This is the result of non-ideal connections between the joints and bricks in the real model of pillars.

The comparison of the propagation velocities, depending on the wave transition through one, two or three mortar joints, is shown in Table 7. The same wave paths as those in the numerical calculations were assumed. The velocities obtained for each pillar differed due to non-ideal connections between the mortar and bricks. Differences between the velocity of the waves passing through one, two, and three joints were also observed. As in the case of the FEM simulations, the velocity value decreased with the increase of the number of joints on the wave path.

## 5. Conclusions

In this study, the ultrasonic tomography technique was applied to the non-destructive diagnostics of masonry pillars. Experimental and numerical investigations were performed on four laboratory specimens: one intact pillar and three pillars with inclusions. The conducted investigations focused on the tomographic velocity reconstruction and the assessment of the internal structure of the pillars.

The study of ultrasonic tomography applied to the assessment of masonry pillars led to the following conclusions:

1. The increase of the number of pixels and paths did not guarantee an improvement of the quality of the tomographic images. In numerical simulations made for the pillar models with idealized connections between the mortar and bricks, more accurate tomograms were obtained when a larger amount of measurement data was collected with a denser division in cells. This observation has not been confirmed in experimental tests conducted on real specimens, with connections between bricks and mortar influenced by air voids and non-ideal adhesion.
2. The change in the velocity value was observed depending on the number of joints through which the wave passed. The velocity decreased with the increase of the number of joints on the wave path.
3. Detection of the arrangement of joints in the cross-section was possible. The joints could be observed, based on numerical data, as line patterns with lower velocities. However, in experimental tests, the increase of the number of paths resulted in the appearance of a large zone, with lower velocity values in the central part of the pillar due to the accumulation of joints and paths crossing them.
4. The inclusion in the form of a hole was identified in both the numerical and experimental tests as an area with lower velocity values.
5. The inclusion in the form of an embedded bar was identified in the numerical data as an area with higher velocity values. This observation was not confirmed in experimental tests due to the existence of many factors that slowed the speed of the wave.
6. The experimental tests enabled the assessment of the quality of the adhesive connection between a steel reinforcing bar embedded inside pillars, and the surrounding pillar body. The tomograms obtained for the pillar with the bar grouted by gypsum mortar revealed similar patterns as those for the pillar with the hole, which indicated poor adhesion. The maps obtained for the pillar with the bar grouted by cement mortar were similar to those obtained for the intact pillar, which indicated strong adhesion.

To summarize, ultrasonic tomography appeared to be an effective technique for the reconstruction of the internal structure of brick pillars. The presented approach may be particularly useful in the diagnostics of applied strengthening and the assessment of the compatibility of reinforcement materials with brick structures, and it may become the basis for the strategic planning of repair procedures. Further investigations should consider the tomographic imaging of masonry structures, accessible from one or two sides, to develop efficient tomographic procedures based on a limited number of inputs and examined ray paths.

## Figures and Tables

**Table 1:** Mechanical properties of the bricks and mortar used for the test specimens.

Material	Density [kg/m^3^]	Young’s Modulus [GPa]	Poisson’s Ratio [-]
brick	1642.04	10.55	0.20
cement mortar	1719.10	8.53	0.15
gauged (cement lime) mortar	1799.90	8.20	0.15
gypsum mortar	1185.70	3.70	0.15

**Table 2:** Numerical wave propagation velocities for configuration #A.

Pillar	Arrangement	v_min_ [m/s]	v_max_ [m/s]	Δv = v_max_ − v_min_ [m/s]	v_avg_ [m/s]	SD [m/s]	CV [%]
#1	A1	2699.52	2750.93	51.41	2732.93	22.0	0.81
	A2	2699.52	2765.47	65.95	2736.24	21.0	0.77
	A3	2681.93	2767.02	85.09	2730.40	20.4	0.75
#2	A1	2692.63	2751.52	58.89	2733.03	23.2	0.85
	A2	2640.62	2766.05	125.44	2731.99	27.7	1.02
	A3	2640.62	2766.05	125.44	2721.84	28.8	1.06
#3	A1	2700.46	2753.27	52.81	2738.87	17.4	0.64
	A2	2700.46	2822.93	122.47	2742.47	22.0	0.80
	A3	2682.24	2825.21	142.97	2737.58	23.0	0.84
#4	A1	2700.46	2753.27	52.81	2738.87	17.4	0.64
	A2	2700.46	2822.93	122.47	2742.49	21.9	0.80
	A3	2682.24	2825.21	142.97	2737.58	23.0	0.84

**Table 3:** Numerical wave propagation velocities for configuration #B.

Pillar	Arrangement	v_min_ [m/s]	v_max_ [m/s]	Δv = v_max_ − v_min_ [m/s]	v_avg_ [m/s]	SD [m/s]	CV [%]
#1	B1	2699.52	2750.93	51.41	2737.67	17.4	0.64
	B2	2693.96	2756.58	62.62	2737.61	17.2	0.63
	B3	2667.36	2771.10	103.74	2727.88	20.9	0.77
#2	B1	2692.63	2751.52	58.89	2735.22	18.8	0.69
	B2	2647.13	2757.36	110.23	2734.08	22.5	0.82
	B3	2618.33	2771.67	153.34	2719.18	29.0	1.07
#3	B1	2700.46	2810.41	109.95	2745.15	21.5	0.78
	B2	2696.75	2810.41	113.66	2745.48	21.4	0.78
	B3	2666.05	2825.21	159.15	2735.68	24.5	0.90
#4	B1	2700.46	2810.41	109.95	2745.15	21.5	0.78
	B2	2696.75	2810.41	113.66	2745.48	21.4	0.78
	B3	2666.05	2825.21	159.15	2735.68	24.5	0.90

**Table 4:** The influence of the number of joints on the numerical wave propagation velocities.

Pillar	v_avg_ [m/s] 1 joint	v_avg_ [m/s] 2 joints	v_avg_ [m/s] 3 joints
#1	2747.70	2733.58	2726.77
#2	2747.19	2734.16	2727.26
#3	2747.45	2734.48	2727.47
#4	2747.45	2734.48	2727.47

**Table 5:** Experimental wave propagation velocities for configuration #A.

Pillar	Arrangement	v_min_ [m/s]	v_max_ [m/s]	Δv = v_max_ − v_min_ [m/s]	v_avg_ [m/s]	SD [m/s]	CV [%]
#1	A1	1687.58	2651.26	963.68	2099.66	363.7	17.32
	A2	1687.58	2651.26	963.68	2104.60	296.1	14.07
	A3	1574.48	2651.26	1076.78	2030.73	258.4	12.72
#2	A1	1253.62	2908.28	1654.66	2100.92	463.0	22.04
	A2	1253.62	2908.28	1654.66	2042.58	395.5	19.36
	A3	1253.62	2908.28	1654.66	1927.77	322.5	16.73
#3	A1	1402.37	2615.69	1213.32	1936.08	304.9	15.75
	A2	1313.12	2615.69	1302.58	1876.67	310.2	16.53
	A3	1150.91	2615.69	1464.78	1731.45	291.4	16.83
#4	A1	1617.59	3068.45	1450.86	2163.11	465.7	21.53
	A2	1617.59	3068.45	1450.86	2107.37	365.2	17.33
	A3	1511.52	3068.45	1556.93	2009.67	316.0	15.72

**Table 6:** Experimental wave propagation velocities for configuration #B.

Pillar	Arrangement	v_min_ [m/s]	v_max_ [m/s]	Δv = v_max_ − v_min_ [m/s]	v_avg_ [m/s]	SD [m/s]	CV [%]
#1	B1	1597.71	2651.26	1053.55	2014.90	330.1	16.38
	B2	1493.14	2651.26	1158.12	1969.88	283.3	14.38
	B3	1321.90	2651.26	1329.36	1896.30	254.4	13.42
#2	B1	1253.62	2908.28	1654.66	1887.48	422.8	22.40
	B2	1117.83	2908.28	1790.45	1807.67	362.3	20.04
	B3	1111.81	2908.28	1796.47	1722.46	311.5	18.08
#3	B1	1222.19	2615.69	1393.51	1814.67	314.8	17.35
	B2	1222.19	2615.69	1393.51	1748.82	314.9	18.01
	B3	1056.88	2615.69	1558.81	1635.34	277.0	16.94
#4	B1	1407.43	3068.45	1661.02	2055.87	424.4	20.64
	B2	1407.43	3068.45	1661.02	2001.88	378.7	18.92
	B3	1354.30	3068.45	1714.15	1921.07	315.8	16.44

**Table 7:** The influence of the number of joints on the experimental wave propagation velocities.

Pillar	v_avg_ [m/s] 1 joint	v_avg_ [m/s] 2 joints	v_avg_ [m/s] 3 joints
#1	2082.75	1727.25	1682.13
#2	1967.77	1657.43	1638.79
#3	1923.85	1578.54	1557.31
#4	2002.48	1800.36	1708.91
